# LGR5 rs17109924 is a predictive genetic biomarker for time to recurrence in patients with colon cancer treated with 5-fluorouracil-based adjuvant chemotherapy

**DOI:** 10.1038/tpj.2015.2

**Published:** 2015-02-10

**Authors:** J Szkandera, S Herzog, M Pichler, V Stiegelbauer, M Stotz, R Schaberl-Moser, H Samonigg, M Asslaber, S Lax, G Leitner, W Renner, H-J Lenz, A Berghold, A Gerger

**Affiliations:** 1 Division of Clinical Oncology, Department of Medicine, Comprehensive Cancer Center Graz, Medical University of Graz, Graz, Austria; 2 Institute for Medical Informatics, Statistics and Documentation, Medical University of Graz, Graz, Austria; 3 Institute of Pathology, Medical University of Graz, Graz, Austria; 4 Department of Pathology, General Hospital Graz West, Graz, Austria; 5 Department of Pathology, General Hospital of Leoben, Leoben, Austria; 6 Clinical Institute of Medical and Laboratory Diagnostics, Medical University of Graz, Graz, Austria; 7 Division of Clinical Oncology, Norris Comprehensive Cancer Center, University of Southern California, Los Angeles, CA, USA

## Abstract

We recently found variants in cancer stem cell genes (*CD44*, *ALCAM* and *LGR5*) significantly associated with increased time to recurrence (TTR) in patients with stage III and high-risk stage II colon cancer treated with 5-fluorouracil (5-FU)-based chemotherapy. In this study, we validated these genetic biomarkers in a large and independent patient cohort (*n*=599). Patients who received 5-FU-based adjuvant chemotherapy (*n*=391) carrying at least one C allele in LGR5 rs17109924 had a significantly increased TTR compared with patients carrying the homozygous T/T variant (HR 0.38, 95%CI 0.19–0.79; *P*=0.006). In patients treated with surgery alone (*n*=208), no association between LGR rs17109924 and TTR was found (*P*=0.728). In the multivariate Cox-analysis, LGR5 rs17109924 remained statistically significant (HR 0.38, 95%CI 0.18–0.78; *P*=0.008) for patients who received adjuvant chemotherapy. We confirmed in a large and independent study cohort that LGR5 rs17109924 is a predictive genetic biomarker for TTR in patients with colon cancer treated with 5-FU-based adjuvant chemotherapy.

## Introduction

Colon cancer is the second leading cause of cancer mortality in Europe and the third cause of cancer-related death in the United States. Approximately 50% of patients with colon cancer develop synchronous or metachronous metastases. The 5-year survival rate of colon cancer patients with metastatic disease is <10%.^1, 2^

Tumor recurrence after curative surgery remains a major issue for improving overall cancer survival. The risk of relapse can be significantly reduced by treating patients with stage III and high-risk stage II colon cancer with 5-fluorouracil (5-FU)-based chemotherapy. However, currently the majority of colon cancer patients receive adjuvant treatment with no benefit, either because they were cured by surgery alone or because they will relapse despite adjuvant treatment. Therefore, there is an urgent need for biomarkers to guide adjuvant treatment strategies for colon cancer patients and improve clinical outcome through patient classification.^3, 4, 5^ Growing evidence indicates that human cancers are stem cell diseases.^6, 7^ A subpopulation of unique neoplastic cells with properties of stem cells within the tumor, known as cancer stem cells (CSCs), possesses the ability to self-renewal and to undergo multilineage differentiation into progenitor cells through asymmetric cell divisions.^6, 7, 8, 9, 10^ Recent data support the existence of CSCs in colon cancer.^11, 12^ These CSCs might be capable of initiating colon cancer development, progression and chemoresistance.^6, 7, 8, 9, 10, 11, 12, 13^ The impact of germline polymorphisms on cancer outcome and chemoresistance is a burgeoning field of research.^14, 15, 16^ There is substantial germline genetic variability within the genes used as markers to identify colon CSCs, including multiple single-nucleotide polymorphisms (SNPs). These common DNA-sequence variations may result in altered gene function and/or activity including transcription, translation or splicing, thereby causing inter-individual differences in patient's tumor recurrence capacity and chemoresistance.^17^

In a recent study, we investigated 25 germline polymorphisms in a comprehensive panel of genes that have been previously associated with colon CSCs to predict tumor recurrence in 234 patients with stage III and high-risk stage II colon cancer treated with 5-FU-based chemotherapy. We found the minor alleles of CD44 rs8193 C>T, ALCAM rs1157 G>A and LGR5 rs17109924 T>C significantly associated with increased time to recurrence (TTR).^18^ However, as all patients included in this preliminary study have been treated with adjuvant chemotherapy, it was not possible to correlate the genotypes with clinical outcome in an untreated control group. Therefore, the aim of the present study was to validate CD44 rs8193 C>T, ALCAM rs1157 G>A and LGR5 rs17109924 T>C based on our preliminary findings in a large and independent study cohort of stage II and III colon cancer patients treated with surgery plus 5-FU-based adjuvant chemotherapy or surgery alone.

## Materials and Methods

### Eligible patients

Between 1995 and 2011, 742 patients with histopathologically confirmed stage II and III colon cancer have been consecutively recruited at the Division of Clinical Oncology, Department of Internal Medicine, Medical University of Graz. The clinical stage according to UICC has been assessed based on the resection specimen and the clinical data at the time of surgery. From 599 patients paraffin-embedded normal tissue adjacent to tumor samples was available for germline genetic testing. A total of 391 patients were treated with adjuvant 5-FU-based chemotherapy and 208 patients were treated with surgery alone. All patients were included in a colon cancer surveillance program, providing history and physical examination and CEA determination every 3 months for 3 years, every 6 months at years 4 and 5 and yearly at years 6–10 after surgery, colonoscopy at year 1 and thereafter every 3–5 years and x-ray of the chest and abdominal ultrasound or CT scans of chest and abdomen every 3–6 months for the first 5 years and x-ray of the chest and abdominal ultrasound yearly from year 6 to10. Patient data were collected retrospectively through chart review. This study has been approved by the Institutional Review Board (IRB) of the Medical University of Graz. All participants were Caucasians.

### Isolation of genomic DNA and determination of SNPs

Tissue samples have been stored at the Biobank of the Medical University of Graz (certified according to EN/ISO 9001:2008) and the archives of the Department of Pathology of the General Hospital Graz West and the General Hospital Leoben (certified according to EN/ISO 9001:2008). Genomic DNA was extracted from paraffin-embedded normal tissue distant from the tumor to obtain germline DNA. Usually the samples from the resection margins were used, and all tissue samples were re-evaluated by a board certified pathologist to ensure tumor-free tissue. DNA isolation was performed using the QIAamp DNA mini Kit (Qiagen, Hilden, Germany) according to the manufacturer's instructions. Genotypes for CD44 rs8193 C>T (3′UTR region), ALCAM rs1157 G>A (3′UTR region) and LGR5 rs17109924 T>C (nonsynonymous coding region) were centrally determined by 5′-exonuclease assay (TaqMan). Primer and probe sets were designed and manufactured using Applied Biosystems ‘Assay-by-Design' custom service (Applera, Vienna, Austria). General TaqMan reaction conditions were documented according to the manufacturer of the assays. End-point fluorescence was measured in a Lambda Fluoro 320 plus plate reader (MWG Biotech AG, Ebersberg, Germany) using excitation/emission filters of 485/530 and 530/ 572 nm, respectively. The data were exported into Excel format and depicted and analyzed as scatter plots. In the plots, genotype groups were identified as separate and distinguishable clusters. As a control for consistency of the genotyping method, determination of genotypes was repeated in at least 96 samples. The rules of good laboratory and clinical practice were observed. The investigator analyzing the germline polymorphisms was blinded to the clinical data set.

### Statistical analysis

The primary end point of the study was TTR, which was defined as the time from date of surgery of colon cancer to the date of first tumor recurrence. If a patient had not recurred, TTR was censored at the time of death or at the last follow-up. On the basis of preliminary findings a hazard ratio (HR) of 0.7 could be detected with a power of 80% and a significance level of 0.0167 (Bonferroni correction applied) across the range of minor allele frequencies (0.2–0.5) using a dominant model. The secondary end point was overall survival (OS), defined as the time from date of surgery of colon cancer to death from any cause. All analyses were performed for the group of patients treated with 5-FU-based adjuvant chemotherapy and for the patients with surgery alone. Allelic distribution of polymorphisms was tested for deviation from Hardy–Weinberg equilibrium using *χ*^2^-test. The association of polymorphisms with TTR and OS was analyzed using Kaplan–Meier curves and compared by log-rank test. In a stepwise backward multivariate Cox-regression analysis, the features sex, age, clinical stage, number of resected lymph nodes and lymphovascular-, vascular- and perineural invasion were included. HR and 95% confidence intervals are reported. Case-wise deletion for missing polymorphisms was used in univariate and multivariate analyses. All analyses were performed using SPSS for Windows (Version 21, SPSS Inc., Chicago, IL, USA).

## Results

The baseline characteristics of the patients included in the analysis and their association with TTR and OS are presented in Table 1. The genotyping quality control provided a genotype concordance of ⩾99%. Genotyping was successful in at least 81.2% of cases for each polymorphism analyzed. In failed cases, genotyping was not successful because of limited quantity and/or quality of extracted genomic DNA. The allelic frequencies for all polymorphisms were within the probability limits of Hardy–Weinberg equilibrium (data not shown).

**Figure 1:**
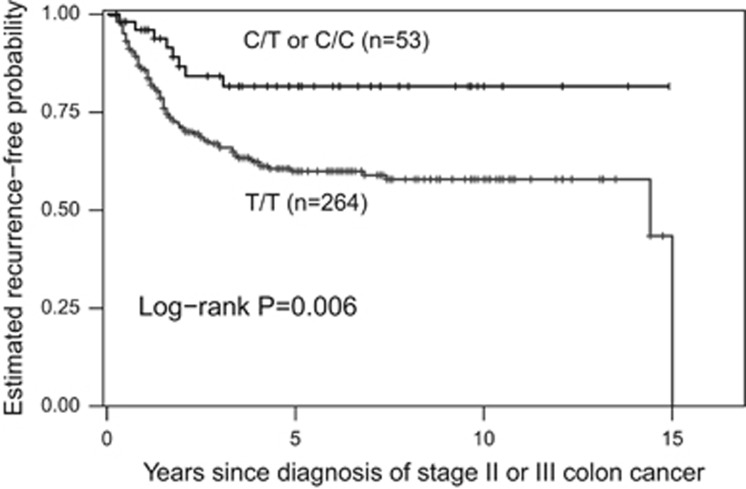
TTR by LGR5 rs17109924 T>C in patients with curative surgery and adjuvant chemotherapy. TTR, time to recurrence.

**Figure 2:**
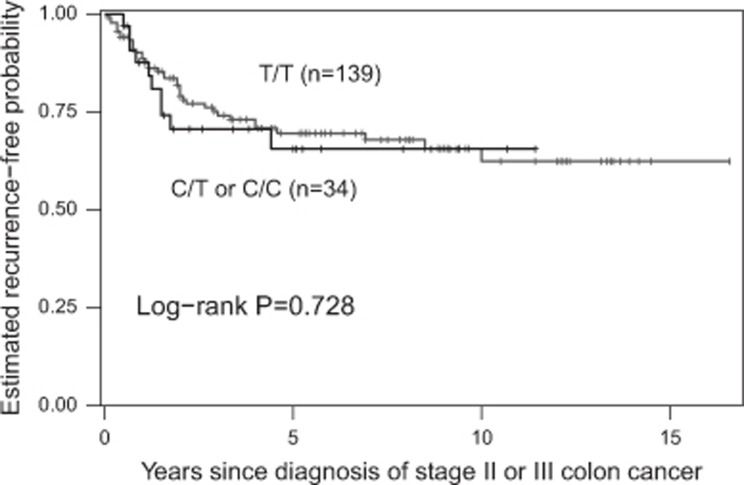
TTR by LGR5 rs17109924 T>C in patients with curative surgery alone. TTR, time to recurrence.

The mean age of the 391 patients receiving adjuvant chemotherapy at the time of diagnosis was 62 years (s.d., 11), with a median follow-up time of 4.2 years (range 0.2–16.2). Tumor recurrence was observed in 127 (32.5%) patients with a stage III- and stage II-dependent probability of 3-year recurrence of 35.8% (s.d., 3.0) and 15.7% (s.d., 4.4), respectively. The tested gene variants CD44 rs8193 and ALCAM rs1157 did not show a statistically significant association with TTR in the analyses (Table 2). Patients with surgery plus 5-FU-based adjuvant chemotherapy carrying at least one C allele in LGR5 rs17109924 had a significantly increased TTR compared with patients carrying the homozygous T/T variant (HR 0.38, 95%CI 0.19-0.79; *P*=0.006). In patients with surgery alone, no association between LGR rs17109924 and TTR was found (*P*=0.728). Patients with adjuvant chemotherapy carrying at least one C allele had a probability of 3-year recurrence of 15.8% (s.d., 5.5). In contrast, patients with the homozygous T/T variant had a probability of 3-year recurrence of 34.0% (sd 3.1; Figure 1). Patients with surgery alone harboring at least one minor allele had a probability of 3-year recurrence of 29.4% (s.d., 8.3) compared with 25.9% (s.d., 4.1) for patients harboring the homozygous T/T variant (Figure 2). Also after adjusting for sex, age, clinical stage, number of resected lymph nodes, lymphovascular, vascular and perineural invasion in a multivariate Cox-regression analysis, LGR5 rs17109924 remained statistically significant for TTR (HR 0.38, 95%CI 0.18–0.78; *P*=0.008) in patients with surgery plus 5-FU-based adjuvant chemotherapy (Table 2). No significant associations were found between the polymorphisms and OS (Table 3).

A total of 391 patients were treated with adjuvant chemotherapy, 272 (69.6%) received 5-FU or capecitabine as monotherapy and 103 (26.3%) received FOLFOX or XELOX regimens. Sixteen (4.1%) patients received various types of adjuvant chemotherapy within clinical trials and were excluded for this analysis. In an explorative analysis, LGR5 rs17109924 was statistically significant for TTR in univariate and multivariate analysis only for patients receiving 5-FU or capecitabine monotherapy. No association was found between LGR5 rs17109924 and TTR for FOLFOX or XELOX (Table 4).

## Discussion

In the present study, we aimed to validate the single biomarkers CD44 rs8193 C>T, ALCAM rs1157 G>A and LGR5 rs17109924 T>C based on our preliminary findings in a large and independent study cohort of 742 stage II and III colon cancer patients. We were able to confirm that LGR5 rs17109924 is a predictive genetic biomarker for TTR in patients with colon cancer treated with 5-FU-based adjuvant chemotherapy.

LGR5, also referred to as GPR49, was originally isolated as a leucine-rich, orphan G-protein-coupled seven-transmembrane receptor that belongs to the G-protein-coupled receptor (GPCR) family of proteins and is a target of Wnt signaling.^19, 20^ In 2007, Barker *et al.*^21^ reported an exclusive expression of LGR5 in cycling columnar cells at the crypt base of the small intestine and that a single LGR5-positive crypt base columnar cell is capable of regenerating all epithelial lineages over a 60-day period. Their findings provide the characterization of the intestinal stem cell by lineage tracing using the expression of a single marker gene, *LGR5*.^21^ In another study, Barker *et al.*^21^ demonstrated in a mouse model that LGR5-positive stem cells are the cells of origin of intestinal tumors by introducing adenomatous polyposis coli mutations into LGR5-positive stem cells, which stimulated adenoma formation in the small intestine and colon.^22^ These data suggest that LGR5 has an important role in colon cancer tumorigenesis. Furthermore, it was reported that the shift in the distribution of LGR5-positive cells toward the lower crypt and/or invasive tumor front might be crucial for the development and progression of colorectal cancer (CRC).^23^ LGR5 has also been detected in tumor spheres derived from colon CSCs.^24^ It has been suggested that, in addition to a marker of intestinal stem cells, LGR5 is an ideal marker for CSC in CRC.^23, 25, 26^ Previous studies showed that LGR5 is overexpressed in hepatocellular carcinoma, ovarian cancer, basal cell carcinoma and esophageal adenocarcinoma.^27, 28, 29, 30^ Using quantitative real-time (RT) PCR, Uchida *et al.*^31^ found that LGR5 mRNA was also frequently overexpressed in colon cancer cell lines. LGR5 expression was found to be higher in colon cancer cell lines derived from metastatic tumors compared with those from primary tumors and correlated significantly with lymphatic invasion, vascular invasion, tumor invasion, lymph node metastasis and clinical stage. Similar results were described by Wu *et al.*^32^ showing that high expression levels of LGR5 receptors were usually associated with more biologically aggressive, advanced and metastatic tumors and that LGR5 is related to worse prognosis in patients with CRC.^32^

Our previous study investigating germline polymorphisms in genes associated with colon CSCs showed that the minor allele of LGR5 rs17109924 T>C independently predicted increased TTR in colon cancer patients treated with 5-FU-based chemotherapy.^18^ LGR5 rs17109924 T>C represents a non-synonymous SNP and was predicted to affect splicing regulation and protein coding by Functional-Single-Nucleotide Polymorphism (F-SNP) database.^33, 34^ Taking into account the clinical association found in our study and the predicted function by F-SNP, we hypothesized that the LGR5 rs17109924 wild-type genotype is associated with higher protein expression of LGR5 leading to a lower TTR. Supporting the biological function and clinical effect of LGR5 rs17109924 T>C, Kleist *et al.*^35^ found a significantly lower immunohistochemical LGR5 expression and a longer TTR in patients with LGR5 variant alleles compared with LGR5 wild-type in stage III CRC. Hsu *et al.*^36^ reported that elevated LGR5 expression levels were significantly associated with advanced clinicopathological features of CRC, including advanced clinical stage and distant metastasis. Furthermore, elevated LGR5 expression levels were associated with shorter disease-free survival, cancer-specific survival and worse treatment response in CRC patients.^36^ Depletion of LGR5 in cultured CRC cells reduced their growth rates and colony formation capability, and enhanced their apoptosis and sensitivity toward 5-FU-based chemotherapy, whereas overexpression of LGR5 increased cell proliferation and reduced the sensitivity of the CRC cells to cytotoxic agents.^36^ Subgroup analysis based on specific 5-FU-based regimens showed that patient with a high LGR5 expression had a trend toward poor response to FOLFOX and FOLFIRI, but all patients with 5-FU monotherapy were non-responder.^36^ Recently, Liu *et al.*^37^ found that the forced expression of LGR5 level increased spheroid formation capacity and spheroid size in CRC cell lines, while decreased LGR5 expression substantially suppressed spheroid formation and renewal in cultured CRC cells, suggesting that LGR5 has a key role in promoting stem-like property.^37^ They also investigated the molecular mechanisms underlying LGR5-associated chemoresistance in CSCs derived from cultured CRC spheroids and showed that elevated LGR5 levels protected spheroids from chemotherapy-induced cell death and increased their resistance to 5-FU and oxaliplatin. Furthermore, they found that the LGR5-associated chemoresistance correlated with elevated expression of ABCB1, a well-characterized efflux pumps for chemotherapeutic drugs.^37^ ABCB1 (also known as MDR1 or p-glycoprotein) belongs to the superfamily of ATP binding cassette (ABC) proteins, which are located in the plasma membranes of cells and in the membranes of cellular organelles.^38, 39^ It transports structurally different hydrophobic chemotherapeutic agents outward in an energy-dependent manner, hence lowering their intracellular concentration and preventing cancer cells from damage by drugs.^40, 41, 42^ This mechanism might elucidate the findings of our study, indicating that the LGR5 rs17109924 T>C wild-type genotype is associated with a higher LGR5 expression, leading to an elevated expression of ABCB1 and resulting in an increased resistance to 5-FU-based chemotherapy. In line with the work by Hsu *et al.*,^36^ in subgroup analysis of our study, LGR5 rs17109924 T>C was a predictive biomarker only in patients receiving 5-FU or capecitabine monotherapy but not for patients with FOLFOX treatment. This might be contradictory to the ABCB1 hypothesis, as this protein transports also other drugs than 5-FU; however, power analysis of the present study was adjusted for all patients with chemotherapy, and not for further subgroup analyses. Some limitations have to be taken into account for this study. Because of the retrospective design a selection bias cannot be fully excluded. For a true validation, this genetic biomarker must be included in a prospective trial. Furthermore, the groups of patients with and without adjuvant chemotherapy differ in some features.

In conclusion, we confirmed in a large and independent retrospective study cohort that LGR5 rs17109924 is a predictive biomarker for TTR in patients with colon cancer treated with 5-FU-based adjuvant chemotherapy. If this effect is limited to patients with adjuvant, 5-FU or capecitabine alone needs to be confirmed.

## Figures and Tables

**Table 1:** Baseline patient characteristics and univariate analysis for TTR and OS in patients treated with 5-FU-based chemotherapy (*N*=391) and in patients with surgery alone (*N*=208)

*Parameter*		*Patients with adjuvant chemotherapy (*N*=391)*						*Patients with surgery alone (*N*=208)*					
		N	*%*	*TTR*		*OS*		N	*%*	*TTR*		*OS*	
				*HR (95% CI)*	P*-value*	*HR (95% CI)*	P*-value*			*HR (95% CI)*	P*-value*	*HR (95% CI)*	P*-value*
Age^a^ in years		62	11	1.02 (1.00–1.04)	0.022	1.03 (1.01–1.05)	0.003	68	11	1.03 (1.00–1.06)	0.040	1.10 (1.06–1.13)	<0.001
*Gender*													
*** ***Male		215	55.0	1 (reference)	0.697	1 (reference)	0.302	115	55.3	1 (reference)	0.759	1 (reference)	0.600
*** ***Female		176	45.0	0.93 (0.66–1.33)		0.81 (0.54–1.21)		93	44.7	1.08 (0.65–1.82)		0.88 (0.55–1.42)	

*T*^b^													
T1 and T2		29	7.4	1 (reference)	<0.001	1 (reference)	<0.001	6	2.9				
	T3		261	66.8	2.92 (0.92–9.25)		1.97 (0.62–6.30)		173	83.2	1 (reference)	0.019	1 (reference)	0.087
T4		101	25.8	5.67 (1.76–18.3)		5.80 (1.79–18.79)		29	13.9	2.10 (1.13–3.89)		1.67 (0.93–3.01)		
	
*Grade*														
*** ***G1 and G2		257	65.7	1 (reference)	0.416	1 (reference)	0.049	161	77.4	1 (reference)	0.597	1 (reference)	0.065	
*** ***G3		134	34.3	1.16 (0.81–1.67)		1.49 (1.00–2.22)		47	22.6	1.18 (0.64–2.15)		1.62 (0.97–2.71)		
	
*Lymph node involvement*														
N0		83	21.2	1 (reference)	<0.001	1 (reference)	<0.001	154	74.0	1 (reference)	<0.001	1 (reference)	<0.001	
N1		186	47.6	1.65 (0.91–3.00)		1.91 (0.95–3.84)		43	20.7	2.56 (1.43–4.57)		2.66 (1.56–4.54)		
N2		122	31.2	4.73 (2.65–8.42)		4.89 (2.49–9.64)		11	5.3	6.98 (3.19–15.25)		6.08 (2.81–13.13)		
	
*Clinical stage*														
*** ***II		82	21.0	1 (reference)	0.001	1 (reference)	0.001	153	73.6	1 (reference)	<0.001	1 (reference)	<0.001	
*** ***III		309	79.0	2.68 (1.54–4.67)		2.96 (1.54–5.70)		55	26.4	3.14 (1.86–5.29)		3.14 (1.93–5.09)		
	
*No. of resected lymph nodes*														
⩽12		47	12.0	1 (reference)	0.817	1 (reference)	0.393	37	17.8	1 (reference)	0.719	1 (reference)	0.532	
>12		344	88.0	0.94 (0.57–1.57)		0.80 (0.47–1.34)		171	82.2	1.13 (0.57–2.25)		1.22 (0.65–2.28)		
	
*Tumor location*														
Left-sided colon		236	60.4	1 (reference)	0.275	1 (reference)	0.115	139	66.8	1 (reference)	0.137	1 (reference)	0.199	
Right-sided colon		155	39.6	1.22 (0.86–1.73)		1.38 (0.93–2.04)		69	33.2	1.49 (0.88–2.53)		1.38 (0.84–2.26)		
	
*Lymphovascular invasion*														
*** ***No		265	67.8	1 (reference)	0.022	1 (reference)	0.086	170	81.7	1 (reference)	0.413	1 (reference)	0.389	
*** ***Yes		126	32.2	1.52 (1.06–2.17)		1.43 (0.95–2.14)		38	18.3	1.30 (0.69–2.46)		1.30 (0.72–2.33)		
	
*Vascular invasion*														
*** ***No		348	89.0	1 (reference)	<0.001	1 (reference)	0.006	191	91.8	1 (reference)	0.014	1 (reference)	0.008	
*** ***Yes		43	11.0	2.31 (1.49–3.59)		2.04 (1.22–3.41)		17	8.2	2.45 (1.20–4.98)		2.40 (1.26–4.58)		
	
*Perineural invasion*^c^														
*** ***No		378	96.7	1 (reference)	0.003	1 (reference)	0.216	206	99.0	ND		ND		
*** ***Yes		13	3.3	3.02 (1.48–6.20)		1.77 (0.72–4.34)		2	1.0					

Abbreviations: HR, hazard ratio; ND, not determined; OS, overall survival; TTR, time to recurrence; 95% CI, 95% confidence interval.

a Mean (s.d.).

b For patients with surgery alone T1, T2 and T3 are combined.

c For patients with surgery alone the analysis was not done (perineural invasion yes *N*=2).

**Table 2:** Univariate and multivariate analysis of polymorphisms and TTR

		*Univariate analysis*		*Multivariate analysis*^a^	
	N	*HR (95% CI)*	P*-value*^b^	*HR (95% CI)*	P*-value*^c^
*Curative surgery with adjuvant chemotherapy*					
** **CD44			0.456		0.595
** **C/C	109	1 (reference)		1 (reference)	
** **C/T^d^	148	0.86 (0.58–1.28)		0.90 (0.60–1.34)	
** **T/T^d^	63				
** **ALCAM			0.924		0.699
** **G/G	193	1 (reference)		1 (reference)	
** **G/A^d^	120	0.98 (0.66–1.46)		0.93 (0.62–1.38)	
** **A/A^d^	13				
LGR5			0.006		0.008
** **T/T	264	1 (reference)		1 (reference)	
** **C/T^d^	52	0.38 (0.19–0.79)		0.38 (0.18–0.78)	
** **C/C^d^	1				
*Curative surgery alone*^e^					
** **CD44			0.170		0.187
** **C/C	55	1 (reference)		1 (reference)	
** **C/T^d^	91	1.55 (0.82–2.90)		1.53 (0.81–2.89)	
** **T/T^d^	36				
** **ALCAM			0.690		0.851
** **G/G	123	1 (reference)		1 (reference)	
** **G/A^d^	50	1.13 (0.63–2.02)		1.06 (0.59–1.91)	
** **A/A^d^	10				
** **LGR5			0.728		0.794
** **T/T	136	1 (reference)		1 (reference)	
** **C/T^d^	29	1.13 (0.56–2.27)		1.10 (0.55–2.21)	
** **C/C^d^	5				

Abbreviations: HR, hazard ratio; TTR, time to recurrence; 95% CI, 95% confidence interval.

a Models were adjusted for sex, age, clinical stage, number of resected lymph nodes, lymphovascular, vascular and/or perineural invasion with a stepwise backward procedure.

b Based on log-rank test.

c Based on Wald test within the Cox-regression model.

d In the dominant model.

e Perineural invasion not included for adjustment in the multivariate model as only 2 of 208 had a perineural invasion.

**Table 3:** Univariate and multivariate analysis of polymorphisms and OS

		*Univariate analysis*		*Multivariate analysis*^a^	
	N	*HR (95% CI)*	P*-value*^b^	*HR (95% CI)*	P*-value*^c^
*Curative surgery with adjuvant chemotherapy*					
** **CD44			0.994		0.701
** **C/C	109	1 (reference)		1 (reference)	
** **C/T^d^	148	1.00 (0.63–1.58)		1.10 (0.69–1.74)	
** **T/T^d^	63				
** **ALCAM			0.864		0.631
** **G/G	193	1 (reference)		1 (reference)	
** **G/A^d^	120	0.96 (0.61–1.51)		0.90 (0.57–1.40)	
** **A/A^d^	13				
** **LGR5			0.297		0.213
** **T/T	264	1 (reference)		1 (reference)	
** **C/T^d^	52	0.72 (0.38–1.35)		0.67 (0.35–1.26)	
** **C/C^d^	1				
*Curative surgery alone*^e^					
** **CD44			0.946		0.783
** **C/C	55	1 (reference)		1 (reference)	
** **C/T^d^	91	1.02 (0.61–1.71)		1.08 (0.64–1.82)	
** **T/T^d^	36				
** **ALCAM			0.582		0.659
** **G/G	123	1 (reference)		1 (reference)	
** **G/A^d^	50	1.16 (0.69–1.96)		1.13 (0.67–1.91)	
** **A/A^d^	10				
** **LGR5			0.520		0.889
** **T/T	139	1 (reference)		1 (reference)	
** **C/T^d^	29	1.22 (0.66–2.27)		1.05 (0.56–1.96)	
** **C/C^d^	5				

Abbreviations: HR, hazard ratio; OS, overall survival; 95% CI, 95% confidence interval.

a Models were adjusted for sex, age, clinical stage, number of resected lymph nodes, lymphovascular, vascular and/or perineural invasion with a stepwise backward procedure.

b Based on log-rank test.

c Based on Wald test within the Cox-regression model.

d In the dominant model.

e Perineural invasion not included for adjustment in the multivariate model as only 2 of 208 had a perineural invasion.

**Table 4:** Univariate and multivariate subgroup analysis for the LGR5 polymorphism and TTR

		*Univariate analysis*		*Multivariate analysis*^a^	
	N	*HR (95% CI)*	P*-value*^b^	*HR (95% CI)*	P*-value*^c^
*Adjuvant chemotherapy=5-FU or capecitabine monotherapy*					
LGR5			0.042		0.023
T/T	186	1 (reference)		1 (reference)	
C/T^d^	35	0.46 (0.21–1.00)		0.41 (0.19–0.89)	
C/C^d^	1				
*Adjuvant chemotherapy=5-FU-based combination therapy*					
LGR5			0.111		0.162
T/T	68	1 (reference)		1 (reference)	
C/T^d^	15	0.23 (0.03–1.70)		0.24 (0.03–1.79)	
C/C^d^	0				

Abbreviations: HR, hazard ratio; TTR, time to recurrence; 95% CI, 95% confidence interval.

a Models were adjusted for sex, age, clinical stage, number of resected lymph nodes, lymphovascular, vascular and/or perineural invasion with a stepwise backward procedure.

b Based on log-rank test.

c Based on Wald test within the Cox-regression model.

d In the dominant model.
